# Cyclic dinucleotides (CDNs) reversed T cells tolerance in anti-tumor immunotherapy

**DOI:** 10.1186/2051-1426-3-S2-P268

**Published:** 2015-11-04

**Authors:** Juan Fu, Young Kim, David Kanne, Meredith Leong, Qi Zeng, Rupashree Sen, Todd D Amstrong, Charles G Drake, Thomas Dubensky, Drew Pardoll, Shekhar Gadkare

**Affiliations:** 1 Johns Hopkis Unversity, Baltimore, MD, USA; 2 Johns Hopkins University, Baltimore, MD, USA; 3 Aduro BioTech, Berkeley, CA, USA; 4 Aduro BioTech, Berkely, CA, USA; 5 Johns Hopkins Unversity, Baltimore, MD, USA; 6 Johns Hopins University, Baltimore, MD, USA; 7 Department of Oncology, Sidney Kimmel Comprehensive Cancer Center at Johns Hopkins University,, Baltimore, MD, USA

## 

One important barrier to cancer immunotherapy. We used cyclic dinucleotides (CDNs) and Listeria vaccine and LPS treated HA-specific CD8 tolerance B10. D2 mice. Our results demonstrated T cells IFN-gamma increased in CDNs and Listeria vaccine plus CDNs treatment groups. But CDNs didn't significantly increase Treg cells. The data showed CDNs can reverse established T cell tolerance that improved cancer immunotherapy in the future. We will further explore T cells tolerance in cancer bearing T cell tolerance mice model in vivo.

